# Bioactive Polyurethane–Poly(ethylene Glycol) Diacrylate Hydrogels for Applications in Tissue Engineering

**DOI:** 10.3390/gels10020108

**Published:** 2024-01-29

**Authors:** Yixuan Yuan, Caleb Tyson, Annika Szyniec, Samuel Agro, Tara N. Tavakol, Alexander Harmon, DessaRae Lampkins, Lauran Pearson, Jerald E. Dumas, Lakeshia J. Taite

**Affiliations:** 1 Department of Chemical Engineering, University of Virginia, Charlottesville, VA 22903, USA; 2 Department of Chemical Engineering, Hampton University, Hampton, VA 23668, USA; 3 Department of Biomedical Engineering, University of Virginia, Charlottesville, VA 22908, USA; 4 Joint School of Nanoscience and Nanoengineering, North Carolina Agricultural & Technical State University, Greensboro, NC 27401, USA; jedumas@ncat.edu

**Keywords:** polyurethane, poly(ethylene glycol) diacrylate, hydrogels, photocrosslinking, cell adhesion, tissue engineering, scaffolds

## Abstract

Polyurethanes (PUs) are a highly adaptable class of biomaterials that are among some of the most researched materials for various biomedical applications. However, engineered tissue scaffolds composed of PU have not found their way into clinical application, mainly due to the difficulty of balancing the control of material properties with the desired cellular response. A simple method for the synthesis of tunable bioactive poly(ethylene glycol) diacrylate (PEGDA) hydrogels containing photocurable PU is described. These hydrogels may be modified with PEGylated peptides or proteins to impart variable biological functions, and the mechanical properties of the hydrogels can be tuned based on the ratios of PU and PEGDA. Studies with human cells revealed that PU–PEG blended hydrogels support cell adhesion and viability when cell adhesion peptides are crosslinked within the hydrogel matrix. These hydrogels represent a unique and highly tailorable system for synthesizing PU-based synthetic extracellular matrices for tissue engineering applications.

## 1. Introduction

Ideal biomaterials possess optimal mechanical properties, biocompatibility, and bio-functionality that work in collaboration with the body to provide support during the entire phase of healing. Hydrogels are three-dimensional hydrophilic networks of polymer chains that can absorb and retain large amounts of water within their structure [1,2,3,4]. Hydrogels can be made from natural materials, including decellularized tissue, protein-derived materials (e.g., collagen, fibrin, elastin), and polysaccharide-based materials (e.g., alginate, chitosan), or from synthetic polymers [5,6]. Synthetic hydrogels include monomers such as acrylamides, acrylates, and diols [7] and offer stricter control over material properties. One challenge in hydrogel development is the need to improve mechanical strength and limited biocompatibility [8]; therefore, there has been a great effort to improve the mechanical properties and bio-functionality of hydrogels through the development of hybrid natural/synthetic hydrogel materials [9,10,11]. Such design efforts to improve biocompatibility and flexibility/toughness with tunable chemistry promote the application of hydrogels in multiple research areas, such as wound dressing, drug delivery, tissue engineering, and smart sensing (e.g., temperature, pH, enzymes) [12,13,14,15,16,17,18]. 

The development of polyurethanes (PUs) in biomaterials research is an ever-broadening field, with numerous medical applications, such as vascular grafts [19,20,21] and scaffolds for bone tissue engineering [19,22,23]. Similar to hydrogel chemistry, PU chemistry is versatile and allows interaction with various functional groups, which has promoted the integration of PU and hydrogel chemistries for the development of hybrid materials. PU hydrogels have advanced, tunable, mechanical properties [24], while maintaining the water-holding capacity of traditional hydrogels [19,25,26]. Furthermore, such materials have been developed for the incorporation of biomolecules for enhanced functionality [27,28]. As a result of their wide range of properties, PU hydrogels have been used in applications such as short-term implants, drug delivery vehicles, and wound dressings [29,30,31,32,33,34].

The versatile chemistries of PU hydrogel materials also enable tunable processability. The precise control of mechanical and structural properties in PU hydrogels, particularly in ultraviolet (UV)-curable formulations, has emerged as a key area of investigation. PU acrylates (PUAs) have attracted much attention as UV-curable coatings due to their excellent flexibility, prominent adhesion on substrates, and a variety of adjustable features [35]. This nuanced control over PUA properties has promoted its use in several biomaterial area applications, including as a bioactive ink in bioprinting [31,32]. Mechanical properties can be tailored by varying UV exposure times, allowing for strict control and the tunability of stiffness for tailored applications in coatings and 3D-printable materials [35,36,37,38]. This intersection of PUAs and hydrogels has great potential to inspire novel innovative biomaterials with targeted functionalities and to advance the use of PU as a biomaterial with clinical relevance.

In this study, tunable bioactive (polyethylene glycol) diacrylate (PEGDA) hydrogels containing photocurable PU were fabricated and characterized to confirm material properties and biocompatibility. The incorporation of bioactive moieties within the hybrid PEG–PU hydrogels promoted the adhesion and growth of human cells, confirming their potential for applications in regenerative medicine. The inclusion of biological function and the ease of fabrication of these materials make them ideal candidates for tissue scaffolds that accurately mimic the cellular environment. This study determined that the integration of PU, PEG hydrogels, peptides, and photocurable chemistries can serve as a versatile basis for potential tissue engineering applications.

## 2. Results and Discussion

### 2.1. Preparation of Photocrosslinked PU Films and PEG–PU Hydrogels

Traditional PEG–PU hydrogels are formed through the addition of PEG into PU chains as a soft segment, followed by additional crosslinking, often using toxic catalysts such as dibutyltin dilaurate (DBTDL) or 1,4-diazabicyclo[2.2.2]octane (DABCO) [25,39,40,41,42,43] in the hydrogel precursor solution. In this work, we demonstrate a simple method of incorporating polyurethane into biocompatible and bioactive PEGDA hydrogels without the need for toxic catalysts or comonomers during gelation. Additionally, the design of this simplified method requires a minimal amount of nonaqueous solvent that can be easily removed and does not impact biocompatibility. Our procedure utilizes a well-characterized method of synthesizing PEGDA, resulting in approximately 97% acrylation [44,45,46,47], and a process for PU synthesis that can be tailored to incorporate PEG and bioactive peptides [20,21,48,49,50]. The addition of 2-hydroxyethyl acrylate (HEA) during the final step of PU synthesis led to terminal photoreactive acrylate groups, as confirmed by the presence of the characteristic proton shifts between 5.5 ppm and 6.5 ppm via ^1^H NMR (Supplementary Materials Figures S1 and S2) and the absence of isocyanate end groups at 2250 cm^−1^ by FTIR (Figure S4). Urethane formation was confirmed by the presence of C=O stretches at 1720 cm^−1^ in the FTIR spectra of both polyurethane diacrylate (PUDA) and PUDA-PQ (Figure S4). The peptide-modified PUDA-PQ appeared as yellow compared to the white PUDA due to the addition of the SPQS peptide, which was recovered as a bright orange product following peptide synthesis and purification as a chain extender. 

**Figure 1:**
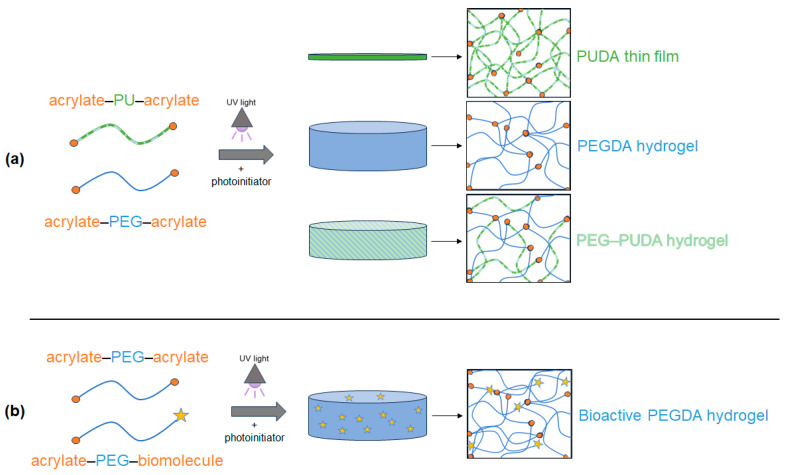
(**a**) Schematic representation of PUDA film, PEGDA hydrogel, and PEG–PUDA hydrogel synthesis under UV light in the presence of the photoinitiator 2,2-dimethoxy-2-phenylacetophenone (DMAP). (**b**) Scheme for the incorporation of bioactive molecules as pendant groups that are crosslinked into hydrogels via conjugation to acrylate-PEG-succinimidyl valerate (acryl-PEG-SVA). Blue and green lines indicate polymer chains, orange dots represent acrylate end groups, and stars represent biomolecules (peptides or proteins).

UV-initiated crosslinking of PUDA and PUDA-PQ without the presence of PEGDA led to the formation of thin PU films (Figure 1). Due to limited solubility of the PUs in THF, we chose to proceed with 5% *w*/*v* and 10% *w*/*v* solutions of PUDA and PUDA-PQ in all experiments. The crosslinking of PEGDA hydrogels, formed using 10% (*w*/*v*) PEGDA, resulted in transparent, water-swollen disks, and the blending of PEGDA with PUDA and PUDA-PQ (Figure 1a) resulted in more opaque disks with noticeably different swelling properties. While this study describes the analysis of PUs that have not been tailored to have increased water solubility, further work with these copolymer hydrogels can utilize previously described methods of including PEG into the PU backbone [21,50], leading to an even wider range of properties for this novel hydrogel system. The reaction of acrylate-PEG-succinimidyl valerate with the cell adhesion peptide RGDS and the angiogenic growth factor VEGF resulted in monoacrylate bioactive polymers that were crosslinked into 3D PEG–PU matrices as pendant moieties that confer bioactivity to our hybrid PEG–PU hydrogels (Figure 1b). 

**Figure 2:**
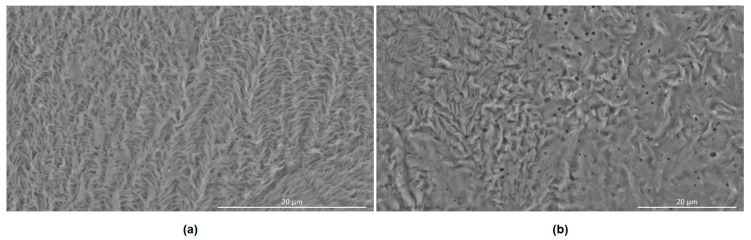
SEM micrographs of (**a**) PEG–PUDA and (**b**) PEG–PUDA-PQ. Scale bars represent 20 μm.

Figure 2 shows scanning electron microscopy (SEM) micrographs of dried samples of PEG–PUDA and PEG–PUDA-PQ. Blended PEG–PU samples exhibit characteristics similar to previously described PEG–PU hydrogels [42], exhibiting a highly crosslinked microstructure. 

### 2.2. Mechanical Testing

**Figure 3:**
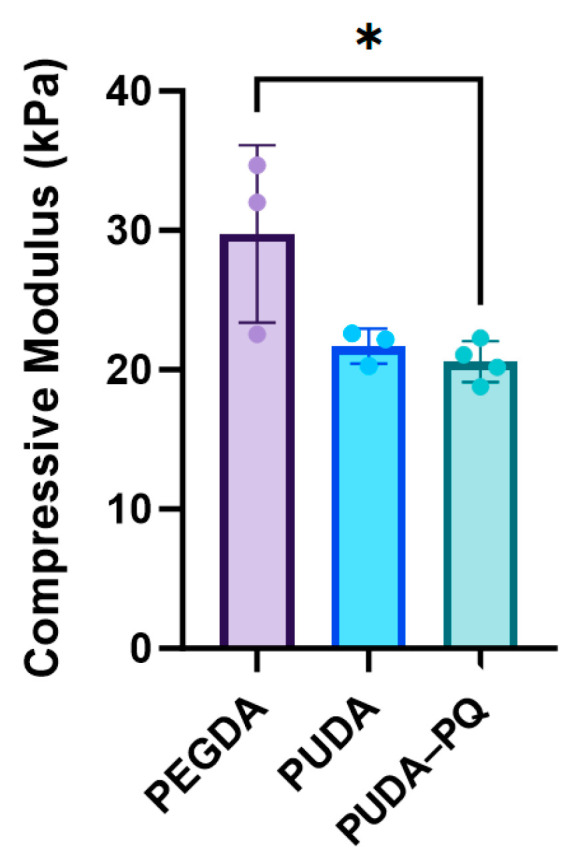
Compressive modulus of hydrogels formed by UV-initiated crosslinking. The modulus was calculated as the slope of the linear region following the toe region of the stress–strain curve. Values reported are the mean ± the standard deviation (*n* = 3); * *p* < 0.05, indicating a statistically significant difference.

Recent studies have shown that the incorporation of varying amounts of PEGDA and vinyl-bearing comonomers, both acrylate [41,42,43,51,52] and allyloxycarbonyl [46,53,54,55], into the hydrogel precursor solution can significantly alter the mechanical properties of UV-cured hydrogels, allowing the fine-tuning of the soft tissue mimetic extracellular matrix (ECM). PEG–PU hydrogels were subjected to compression testing, and the resulting stress–strain curves were analyzed to detect the linear region following the toe region of the collected data (Figure S7). The slope of the linear region was taken as the compressive modulus, as shown in Figure 3. PEGDA hydrogels of similar composition (10% *w*/*v*, 6000 MW) typically display a modulus in the range of tendons or cartilaginous tissues and have been used to simulate the ECM of fibrotic lesions and tumors [51,52,55,56,57], which are stiffer than most soft tissues [41,42,58,59]. Blending PU into PEGDA hydrogels reduced the compressive modulus by more than 25% and complements recent prior studies showing that different species of vinyl-bearing copolymers can have a significant impact on hydrogel mechanical properties. The inclusion of PU into PEGDA hydrogels also resulted in a decrease in the storage modulus (G′) without significantly altering the loss modulus (G″; Figure S8 and Table S1). The ability to tune the mechanical properties of hydrogels based on the addition of equally biocompatible copolymers or comonomers represents a powerful platform to investigate the influence of matrix stiffness on cell behavior in a broad range of biological systems, and these hydrogels show promise in allowing us to tailor synthetic ECM properties.

### 2.3. Hydrogel Swelling and Degradation

**Figure 4:**
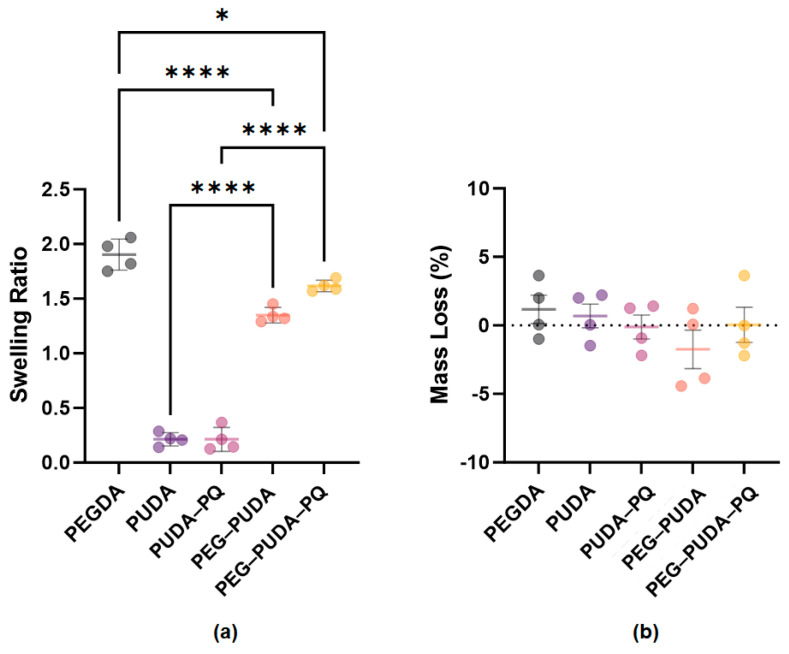
(**a**) Equilibrium swelling ratio of PEGDA hydrogels, PU films, and PEG–PUDA hydrogels in PBS at 37 °C. (**b**) Mass loss of hydrogels and thin films after 14 days in collagenase solution at 37 °C. Error bars indicate the standard deviation from the mean of 4 samples per condition (*n* = 4); * *p* < 0.05, **** *p* < 0.0001.

Swelling behavior is important in the performance of hydrogels as tissue scaffolds, since soft tissues consist of approximately 60–80% water [60,61]. PEGDA is one of the most extensively studied hydrogel systems due to its desirable swelling properties, as well as the ability to fine-tune hydrogel crosslinking density, swelling, degradation, and mechanical properties and the inclusion of biomolecules to stimulate cellular responses [1,2,3,46,62,63,64]. The swelling behavior of PEG–PU hydrogels has been demonstrated to vary based on the amount of crosslinking [41,42,43] and the hydrophilicity of the incorporated PU [42,65]. In assessing the swelling of our photocrosslinked PEG–PU hydrogels and PU films at 37 °C, we observed minimal swelling behavior of both 10% *w*/*v* PUDA (0.213 ± 0.06) and 10% *w*/*v* PUDA-PQ (0.213 ± 0.109) films (Figure 4a). The swelling behaviors of both PEG–PU hydrogels increased significantly compared to PU films (1.53 ± 0.069 for PEG–PUDA and 1.62 ± 0.053 for PEG–PUDA-PQ) yet were also lower than the swelling ratio observed with PEGDA hydrogels (1.90 ± 0.142). These results demonstrate that PUs photocured with PEGDA are a potential means of tailoring the swelling behavior of hydrogels and that inclusion of more hydrophilic segments into PU chains may serve to further tune hydrogel mechanical properties. No significant mass loss was observed in any of the hydrogel and film formulations (Figure 4b).

### 2.4. Cell Adhesion and Viability

**Figure 5:**
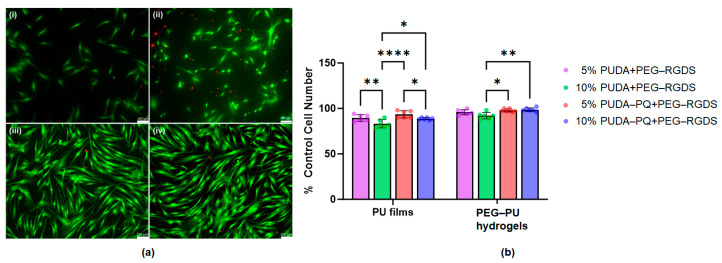
Viability of HDFs after 48 h of culture on thin films and hydrogels containing acryl-PEG–RGDS. (**a**) Representative images of HDFs seeded on (i) 5% *w*/*v* PUDA films, (ii) 5% *w*/*v* PUDA-PQ films, (iii) PEGDA hydrogels containing 5% *w*/*v* PUDA, and (iv) PEGDA hydrogels containing 5% *w*/*v* PUDA-PQ. At least 3 images were taken of each sample, with *n* = 4 per condition. Green fluorescence represents live cells, while red fluorescence indicates dead cells. Scale bars = 100 μm. (**b**) Quantification of live cells on PU films and PEG–PU hydrogels compared to control PEGDA hydrogels containing PEG–RGDS. Error bars represent the standard deviation from the mean of 4 samples per condition (*n* = 4); * *p* < 0.05; ** *p* < 0.01; **** *p* < 0.0001.

Cell adhesion and viability on crosslinked PU films and PEG–PU hydrogels were assessed through the addition of a PEGylated cell adhesion peptide, the fibronectin-derived sequence RGDS (3.5 mM), into the precursor solution for each film or hydrogel. Solutions were sterilized using 0.2 μm filters, and films and hydrogels were crosslinked by UV light. Human dermal fibroblasts (HDFs) were seeded onto the surfaces of either PU films or PEG–PU hydrogels formulated with either 5% *w*/*v* PUDA and PUDA-PQ or 10% *w*/*v* PUDA and PUDA-PQ. Following 48 h of culture on films and hydrogels, cell adhesion and viability were visualized using a LIVE/DEAD viability stain and fluorescence microscopy. HDF adhesion on photocured 5% *w*/*v* PUDA (Figure 5a, panel (i)) and PUDA-PQ films (Figure 5a, panel (ii)) is lower than that observed on 5% *w*/*v* PEG–PUDA (Figure 5a, panel (iii)) and PEG–PUDA-PQ (Figure 5a, panel (iv)) hydrogels, likely due to hindered presentation of the PEG–RGDS within the thin films. Within the water-swollen 3D matrix of the hydrogels, the PEGylated RGDS is grafted into the hydrogel backbone, leaving the adhesion peptide free to interact with cells [3,4,66], which has been shown to increase cell spreading, migration, and survival [45,67,68,69,70,71,72]. HDFs under all conditions show an elongated morphology, indicating cell spreading and significant levels of adhesion through interactions of cell-surface integrins with RGDS.

While cell adhesion was lower on PU films, cell viability remained high on both films and PEG–PU hydrogels (Figure 5b). While there were no statistically significant differences in viability between films and hydrogels, the film and hydrogel formulations did show differences within each group. The films containing higher densities of polyurethane had lower cell viability across both types of PU (89.5 ± 3.62% for 5% PUDA compared to 83.17 ± 4.49% for 10% PUDA and 93.5 ± 3.89% for 5% PUDA-PQ compared to 88.67 ± 1.21% for 10% PUDA-PQ). However, in the hydrogel group, this observation was not the case for all conditions (95.83 ± 2.86% for 5% PUDA compared to 92 ± 3.52% for 10% PUDA and 97.84 ± 1.72% for 5% PUDA-PQ compared to 98.67 ± 2.07 for 10% PUDA-PQ). Though cell viability was above 80% for all conditions, it is possible that the higher density films retained more residual THF after rinsing, which impacted cell viability. The water-swollen hydrogels are more likely to have allowed for most, if not all, of the THF to be rinsed away after consecutive swelling in PBS and cell culture media, resulting in higher cell viability. All conditions are normalized to cell viability on PEGDA hydrogels containing 3.5 mM PEG–RGDS (100 ± 1.29% viability).

**Figure 6:**
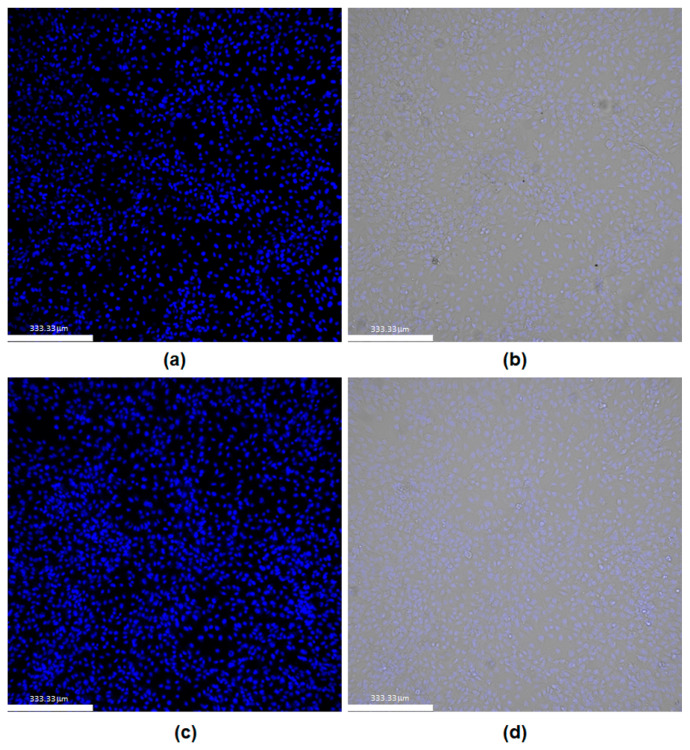
HUVEC viability after 10 days of culture on PEGDA and PEGDA–PUDA hydrogels containing acryl-PEG–RGDS and acryl-PEG–VEGF. NucBlue staining of (**a**) HUVEC nuclei on PEGDA hydrogels, (**b**) merged fluorescent and brightfield images on PEGDA hydrogels, (**c**) HUVEC nuclei on PEG–PUDA hybrid hydrogels, and (**d**) merged fluorescent and brightfield images on PEG–PUDA hydrogels. Scale bars = 333.5 μm.

Additionally, PEG–PUDA hydrogels were assessed for their ability to support the longer-term culture of human umbilical vein endothelial cells (HUVECs). Hydrogel precursor solutions were sterilized using 0.2 μm filters, and hydrogels were swollen for 24 h in PBS, followed by swelling in endothelial cell growth media for another 24 h. HUVECs were seeded on either PEGDA hydrogels containing the adhesion ligand RGDS and the angiogenic growth factor VEGF or on PEG–PUDA hydrogels (10% *w*/*v* PUDA) containing RGDS and VEGF. After 10 days of culture, cell viability was assessed using a NucBlue viability stain, which emits blue fluorescence when bound to DNA. After the 10-day culture period, HUVECs covered both gels in a monolayer (Figure 6), with no observable differences in viability between the two types of hydrogels, indicating that the PEG–PUDA hydrogels were able to sustain HUVEC viability long term in a similar manner to PEGDA hydrogels. Again, cells exhibited a spread morphology, indicating that HUVECs had thoroughly adhered and had the potential to divide and migrate during the 10-day culture period [72]. Further, the successful inclusion of bioactive proteins into these hybrid materials presents us with the opportunity to engineer cell culture microenvironments that more closely resemble the ECM of soft tissues, increasing their potential as scaffolds with the required biochemical and biomechanical properties for 3D tissue growth.

## 3. Conclusions

We have developed a quick and facile method of incorporating an acrylate polyurethane into PEGDA hydrogels, resulting in biocompatible scaffolds with tunable mechanical properties and bioactivity. PEG–PU hydrogels’ compressive moduli, which were lower than those of PEGDA hydrogels, fall into the range of ECM stiffness observed in soft tissues, providing a potentially tunable mechanical range that can be used to study the physiological responses of cells cultured in these hydrogels. The swelling profile of PEG–PU hydrogels also has potential to be tailored as a function of crosslinked PU. While our initial study focused on the feasibility of integrating a largely water-insoluble, nondegradable polyurethane into a UV-crosslinked PEG hydrogel system, this proof-of-concept now allows us to consider further tailoring of our acrylate-PU design to explore the wide range of biochemical and biomechanical properties possible in this hybrid photocurable hydrogel system. The ease with which this system can incorporate a variety of biologically active moieties, such as PEGylated peptides and proteins, was demonstrated, as was the viability of mammalian cells in culture on hybrid polymer hydrogel scaffolds. The successful integration of bioactive peptides in PEG–PU and PEG–PUDA hydrogels promoted the adhesion and subsequent proliferation of HDFs on the hydrogel surfaces. This work provides a step forward in the design of PEG–PU scaffolds for tissue engineering applications that can be strictly tailored to mimic the native ECM environment.

## 4. Materials and Methods

### 4.1. Synthesis of Polyurethane Diacrylate

Diacrylate polyurethane was synthesized by first forming a prepolymer of methylene di(p-phenyl isocyanate) (MDI; Sigma-Aldrich Inc., St. Louis, MO, USA) and poly(tetramethylene oxide) (PTMO; Sigma-Aldrich Inc., St. Louis, MO, USA) and extending the polymer chain with 1,4 butanediol (BD; Sigma-Aldrich Inc., St. Louis, MO, USA) [20,21,48,49,50]. A 10% (*w*/*v*) solution of MDI (4 mmol; MW 250) was dissolved in 10 mL of anhydrous *N,N*-dimethylformamide (DMF) in a 100 mL three-neck round bottom flask and stirred at room temperature. A 10% (*w*/*v*) solution of PTMO (2 mmol; MW 2000) in 20 mL of anhydrous DMF was added, and the mixture was heated to 75 °C and reacted for 3 h under argon. The resulting isocyanate-terminated prepolymer was cooled to room temperature, and BD (2 mmol; MW 90) in 2 mL of anhydrous DMF was added as a chain extender, facilitating chain growth by reaction of the isocyanate groups on the prepolymer with the hydroxyl groups of the diol. Using the method described by Li et al., chain extension was terminated by adding 2-hydroxyethyl acrylate (HEA) as an end-capping reagent [73]. A solution of HEA (4 mmol; MW 116; Sigma-Aldrich Inc., St. Louis, MO, USA) in 2 mL of anhydrous DMF was added dropwise, and the reactor was heated to 45 °C. The reaction proceeded at 45 °C overnight before the solution was cooled to room temperature, precipitated in methanol, and dried. The resulting polymer, referred to as PUDA, was stored protected from light. The PUDA was characterized by ^1^H NMR (Figure S1) using a Bruker Avance III 600-MHz spectrometer with *N,N*-dimethylformamide-d7 as the solvent (Sigma-Aldrich Inc., St. Louis, MO, USA) and by Fourier-transform infrared spectroscopy (FTIR; Bruker Invenio-S; Figure S4).

### 4.2. Synthesis of Peptide-Modified Polyurethane Diacrylate

A model noncell adhesive peptide containing a protease-sensitive domain, SPQGIWGQS (ser-pro-gln-gly-ile-trp-gly-gln-ser; SPQS), was synthesized on a Liberty Blue microwave peptide synthesizer using fluorenylmethoxycarbonyl (FMOC) chemistry (CEM Corporation, Matthews, NC, USA). SPQS was dialyzed against ultrapure water, lyophilized, and characterized by matrix-assisted laser desorption/ionization time-of-flight (MALDI-TOF) spectroscopy. The SPQS peptide was designed to contain hydroxyl groups at both the N- and C-terminal ends through the addition of the amino acid serine, which contains a hydroxymethyl side chain that has been shown to react with terminal isocyanate prepolymers in the same manner as other diols [20,21]. The polyurethane prepolymer was synthesized with MDI and PTMO as described above and was extended with a combination of the SPQS peptide (0.2 mmol) and BD (2 mmol) in 3 mL of anhydrous DMF. HEA was again added dropwise to terminate chain extension, and the reactor incubated at 45 °C overnight under argon. The reactor was then cooled to room temperature, and the resulting polymer precipitated in methanol. The product, PUDA-PQ, was dried and stored protected from light. The PUDA-PQ was characterized by ^1^H NMR (Figure S2) and FTIR (Figure S4).

### 4.3. Synthesis PEGDA and PEG–Peptide and –Protein Conjugates

Polyethylene glycol diacrylate (PEGDA) was synthesized by dissolving 24 g dry PEG (MW: 6000; Sigma-Aldrich Inc., St. Louis, MO, USA) in anhydrous dichloromethane (DCM; Sigma-Aldrich Inc., St. Louis, MO, USA) with an equimolar amount of triethylamine and 1.45 g acryloyl chloride (Sigma-Aldrich Inc., St. Louis, MO, USA), added dropwise. The mixture was then stirred under argon for 24 h, washed with 2 M K_2_CO_3_, and separated into aqueous and DCM phases to remove HCl. The DCM phase was dried by repeated centrifugation to separate and remove any additional water, and the PEG diacrylate was then precipitated in diethyl ether, filtered, and dried under vacuum at room temperature overnight. The resulting polymer was dialyzed overnight against DI water to remove any residual salts and impurities, dissolved in chloroform-d, and characterized by proton NMR to confirm acrylation (Figure S3). The PEGDA was stored under a blanket of argon, protected from light, at −20 °C.

The cell adhesion peptide RGDS (Genscript, Piscataway, NJ, USA) was conjugated to PEG by reacting 2.1 molar equivalents of the heterobifunctional linker acrylate-PEG-succinimidyl valerate (acryl-PEG-SVA; Layson Bio, Arab, AL, USA) in dimethyl sulfoxide (DMSO; Sigma-Aldrich Inc., St. Louis, MO, USA) with 2.1 molar excess *N,N*-diisopropylethylamine (DIPEA; Sigma-Adrich Inc., St. Louis, MO, USA) to acryl-PEG-SVA. The reaction was performed overnight at room temperature under argon. The PEGylated peptide was dialyzed against ultrapure water and lyophilized. The conjugation efficiency and purity of the peptide conjugate were analyzed by matrix-assisted laser desorption/ionization (MALDI) mass spectrometry (Figure S6). The PEG–RGDS conjugates were stored protected from light at −20 °C, under argon.

Vascular endothelial growth factor (VEGF) (400:1 molar ratio, Genscript Biotech, Piscataway, NJ, USA) was conjugated to acryl-PEG-SVA (Layson Bio, Arab, AL, USA) in 50 mM sodium bicarbonate (pH 8.5) at 4 °C for 4 days as previously described by Moon et al. [74]. The resulting mixture was lyophilized in a sterile manner, reconstituted in HEPES buffered saline (HBS, pH 7.4) with 0.1% BSA and stored at 4 °C for up to 3 months. Growth factor PEGylation was assessed by sodium dodecyl-sulfate polyacrylamide gel electrophoresis (SDS-PAGE) and the activity of PEGylated VEGF was assessed by an enzyme-linked immunosorbent assay (ELISA) specific to VEGF (Invitrogen, Carlsbad, CA, USA).

### 4.4. Preparation of PU Films and PEG–PU Hydrogels

PUDA and PUDA-PQ films were synthesized by dissolving each polymer in THF at either 5% or 10% *w*/*v*. The photoinitiator 2-dimethoxy-2-phenylacetophenone (DMAP; Sigma-Aldrich Inc., St. Louis, MO, USA), prepared by dissolving 300 mg DMAP in 1 mL of *N*-vinylpyrrolidone (NVP; Sigma-Aldrich, St. Louis, MO, USA), was then added at 10 μL/mL precursor solution. Glass coverslips previously pretreated with ethanol with 2% (*v*/*v*) 3-(trimethoxysilyl) propyl methacrylate (VWR, Radnor, PA, USA) for 3 days to methacrylate the glass surface were rinsed twice with 70% ethanol then three times with PBS and stored under argon until use. Thin PU films were formed in 6 mm diameter silicon molds (1 mm thickness) adhered to pretreated glass coverslips. Then, 35 µL of PU precursor solution containing DMAP was added to the center of each mold, and each precursor formulation was crosslinked by exposure to long-wave UV light (365 nm, 10 mW/cm^2^) for 30 s. To form cell-adhesive thin films, PEGylated RGDS was also dissolved in THF and added to the film precursor solution to form a 3.5 mM solution of RGDS.

PU–PEG hybrid hydrogels were formed in a similar fashion, with consideration of the water-insoluble nature of the PU polymers. PEGDA was dissolved at 10% *w/v* in HEPES buffered saline (HBS) at pH 7.4. As described above, PU precursors were formed at different dilutions (5%, 10%) to test their compatibility with the PEGDA precursor solution. Upon mixing at higher percentages of PUDA and PUDA-PQ, we observed a small amount of particulate precipitation, but no significant amount of the solubilized PU was noticeable. Hydrogels were formed in the presence of DMAP on glass coverslips as described above and assessed by FTIR for inclusion of PU into the PEGDA hydrogels (Figure S5). Cell-adhesive hydrogels were formed through the addition of 3.5 mM acryl-PEG–RGDS into the PEGDA precursor solution.

SEM was used to investigate the morphology of the blended PEG–PU hydrogels using a Phenom XLG2 environmental scanning electron microscope (ESEM; Thermo Scientfic, Waltham, MA, USA). The instrument has a cerium hexaboride thermionic electron source with a resolution <10 nm (30 kV). Hydrogels were dried for 48 h prior to SEM imaging.

### 4.5. Mechanical Testing

Compression testing was performed on hydrogels swollen overnight in PBS using a Discovery HR Hybrid Rheometer (TA Instruments, New Castle, DE, USA). Hydrogels were compressed at a strain rate of 0.003 mm/s. The resulting stress–strain curves were analyzed to find the slope of the linear region immediately following the toe region, which was taken as the compressive modulus (Figure S7). An Anton Parr MCR 302 rheometer with an 8 mm flat plate geometry was used to characterize in situ gelation mechanics at 25 °C (Figure S8 and Table S1). Hydrogels and films were cured during oscillatory shear time sweeps (1 Hz, 1% strain) with a 2 min UV exposure (365 nm, 10 mW/cm^2^). Precursor solution (25 mL) was pipetted onto the UV-configured plate of the rheometer, and an initial 30 s time sweep was followed by 120 s of UV light exposure and 30 s of continued time sweep following UV light exposure.

### 4.6. Swelling and Degradation Studies

The capacity of crosslinked thin films and hydrogels to swell in aqueous solution was assessed by immersing samples (6 mm diameter) in phosphate-buffered saline (PBS) at 37 °C for 14 days. Each sample’s wet weight was measured over time using an analytical balance until an equilibrium swelling level was reached. The equilibrium gravimetric swelling ratio was then calculated as

(1) $$Swelling\;Ratio=\frac{W_{s}-W_{i}}{W_{i}},$$

where *Ws* is the weight of the hydrogel after swelling, and *Wi* is the initial weight after crosslinking. In addition, since the model peptide incorporated into PUDA-PQ contains a well-characterized enzymatically degradable sequence, we also assessed, in a parallel study, any swelling profile changes when gels and films were incubated in a collagenase solution in PBS (10 mg/mL) at 37 °C for 14 days. The mass loss of hydrogels and films after the incubation period was calculated as

(2) $$\%\;Mass\;Loss=\frac{m_{i,d}-m_{d}}{m_{i,d}}\times 100,$$

where the initial dry mass (*m_i,d_*) of films and hydrogels was determined as the average of vacuum-dried samples formed in the same manner as those incubated in collagenase, and the dry mass after swelling (*m_d_*) was determined after vacuum-drying samples incubated with collagenase for 14 days.

### 4.7. Cell Maintenance

Human dermal fibroblasts (HDFs; American Type Culture Collection, Manassas, VA, USA), passage 4, and human umbilical vein endothelial cells (HUVECs; Lonza, Walkersville, MD, USA), passage 2, were used in this study. HDFs were maintained in Dulbecco’s Modified Eagle Medium (DMEM; Thermo Fisher Scientific, Waltham, MA, USA) supplemented with fetal bovine serum (FBS; Thermo Fisher Scientific, Waltham, MA, USA) and glutamine–penicillin–streptomycin (GPS; Thermo Fisher Scientific, Waltham, MA, USA) at 37 °C in a 5% CO_2_ environment. HUVECs were cultured in Microvascular Endothelial Growth Medium-2 (EGM-2MV; Lonza, Walkersville, MD, USA), supplemented with fetal bovine serum, hydrocortisone, human fibroblast growth factor, vascular endothelial growth factor, insulin-like growth factor, ascorbic acid, human epidermal growth factor, and gentamicin sulfate-amphotericin. HUVECs were maintained in EGM-2MV at 37 °C in a 5% CO_2_ environment.

### 4.8. Cell Adhesion and Viability Studies

HDFs were used to assess the ability of both PUDA and PUDA-PQ to facilitate cell adhesion and maintain cell viability over 48 h of culture. PU thin films and PU–PEG hydrogels were formed adhered to glass-bottom 24-well plates (Cellvis, Mountain View, CA, USA). The well plates were pretreated with ethanol with 2% (*v*/*v*) 3-(trimethoxysilyl) propyl methacrylate for 3 days to methacrylate the glass surfaces, rinsed twice with 70% ethanol, then three times with PBS, and stored under argon until use. Hydrogels and films were formed in 6 mm diameter silicon molds (1 mm thickness) adhered to each well of the pretreated plates. Hydrogel precursor solutions were sterile filtered using a 0.2 μm syringe filter (Pall Corporation, Port Washington, NY, USA), and 35 μL of precursor solution containing DMAP was added to the center of each mold and exposed to UV light to form hydrogel disks or thin crosslinked PU films. The molds containing hydrogel precursor were covered with a glass slide pretreated with Sigmacote (Sigma-Aldrich Inc., Milwaukee, WI, USA) to ensure flat hydrogel surfaces. Hydrogels were then incubated in PBS for 24 h to remove any residual THF and then swollen in DMEM for another 24 h. HDFs were then seeded on hydrogel surfaces at a density of 1 × 10^6^ cells/gel and cultured for 48 h. Following the culture period, cells were stained with a LIVE/DEAD viability/cytotoxicity kit (Invitrogen, Waltham, MA, USA), which uses 1 μM calcein-AM and 4 μM ethidium homodimer-1 to stain live cells green and dead cells red. After a 30 min incubation with the LIVE/DEAD solution, cells were imaged by fluorescence microscopy. The percentage of cells alive on each film or hydrogel were quantified by counting all cells in 3 images per condition and dividing the total number of live (green) cells by the number of total cells per field of view.

Further studies to assess the viability, spreading, and proliferation of cells in longer-term culture on PU–PEG hybrid hydrogels were performed using HUVECs. Hydrogels were formed by combining a 10% *w*/*v* solution of PUDA with a 10% *w*/*v* solution of PEGDA in HEPES buffer and adding 3.5 mM acryl-PEG–RGDS. The solution was then sterile-filtered using a 0.2 μm syringe filter, and 1.9 ng sterile acryl-PEG–VEGF and 10 mL DMAP were added. Upon crosslinking via exposure to UV light, hydrogels were incubated with PBS for 24 h to remove residual THF then swollen in EGM-2MV for an additional 24 h. HUVECs were seeded on the surfaces of the hydrogels at a density of 8 × 10^4^ cells/gel and cultured for 10 days. After the culture period, cells were stained with NucBlue nuclear stain, and fluorescence microscopy was used to visualize cells on hydrogel surfaces. 

### 4.9. Statistical Analysis

A sample size of at least 3 hydrogels or thin films were analyzed in each experiment, and the data are reported as the mean ± the standard deviation. The statistical analyses in this study were conducted using one-way or two-way analysis of variance (ANOVA) with Tukey’s post-hoc test to determine statistical significance, with a minimum significance level of *p* < 0.05.

## Data Availability

The data that support the findings of this study are available from the corresponding author upon request. The data are not publicly available due to ongoing research.

## Supplementary Materials

The following supporting information can be downloaded at https://www.mdpi.com/article/10.3390/gels10020108/s1, Figure S1: NMR spectra of PUDA, Figure S2: NMR spectra of PUDA-PQ, Figure S3: NMR spectra of PEGDA, Figure S4: FTIR spectra of PUDA and PUDA-PQ, Figure S5: FTIR spectra of PEGDA, PEG-PUDA, and PEG-PUDA-PQ hydrogels, Figure S6: MALDI spectra of acrylate-PEG-SVA and acrylate-PEG-RGDS, Figure S7: Stress-strain curves for PEGDA, PEG-PUDA, and PEG-PUDA-PQ hydrogels, Figure S8: Storage and loss modulus for PEGDA, acrylate polyurethane films, and PEGDA-polyurethane hydrogels, Table S1: Rheology data from hydrogels cured during oscillatory shear time sweeps, Table S2: Swelling ratios and percent mass loss of hydrogels and thin PU films.
