# Supplementary material for: Bioactive Polyurethane–Poly(ethylene Glycol) Diacrylate Hydrogels for Applications in Tissue Engineering
Source: Gels. 2024 Jan 29;10(2):108. doi: 10.3390/gels10020108 (PMC10887679; doi:10.3390/gels10020108)
Supplement: Supplementary file 1 [file gels-10-00108-s001.zip › gels-2825486-supplementary.pdf]

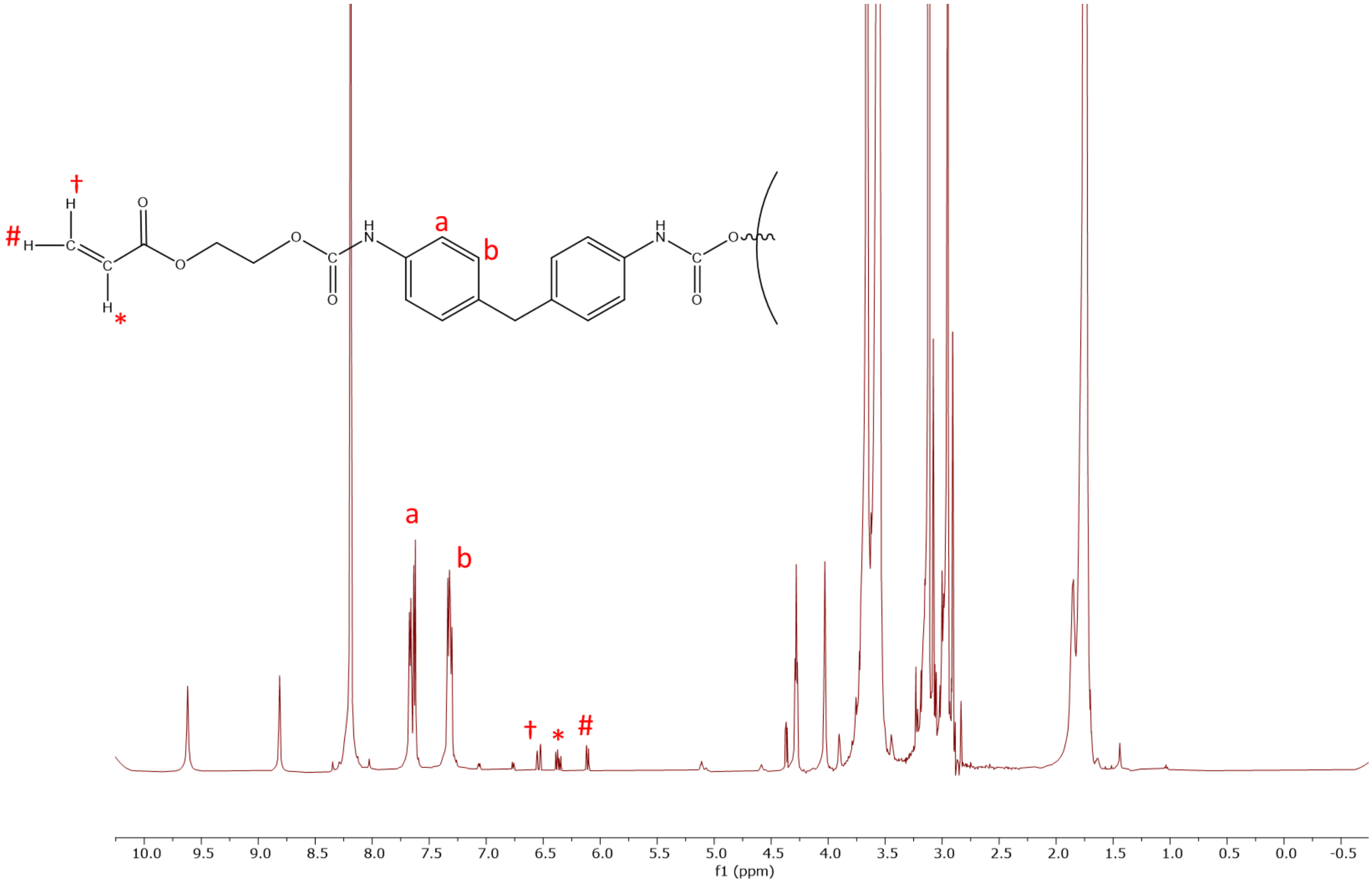

Figure S1. ^{1}H NMR spectra of PUDA in DMF-d_7.

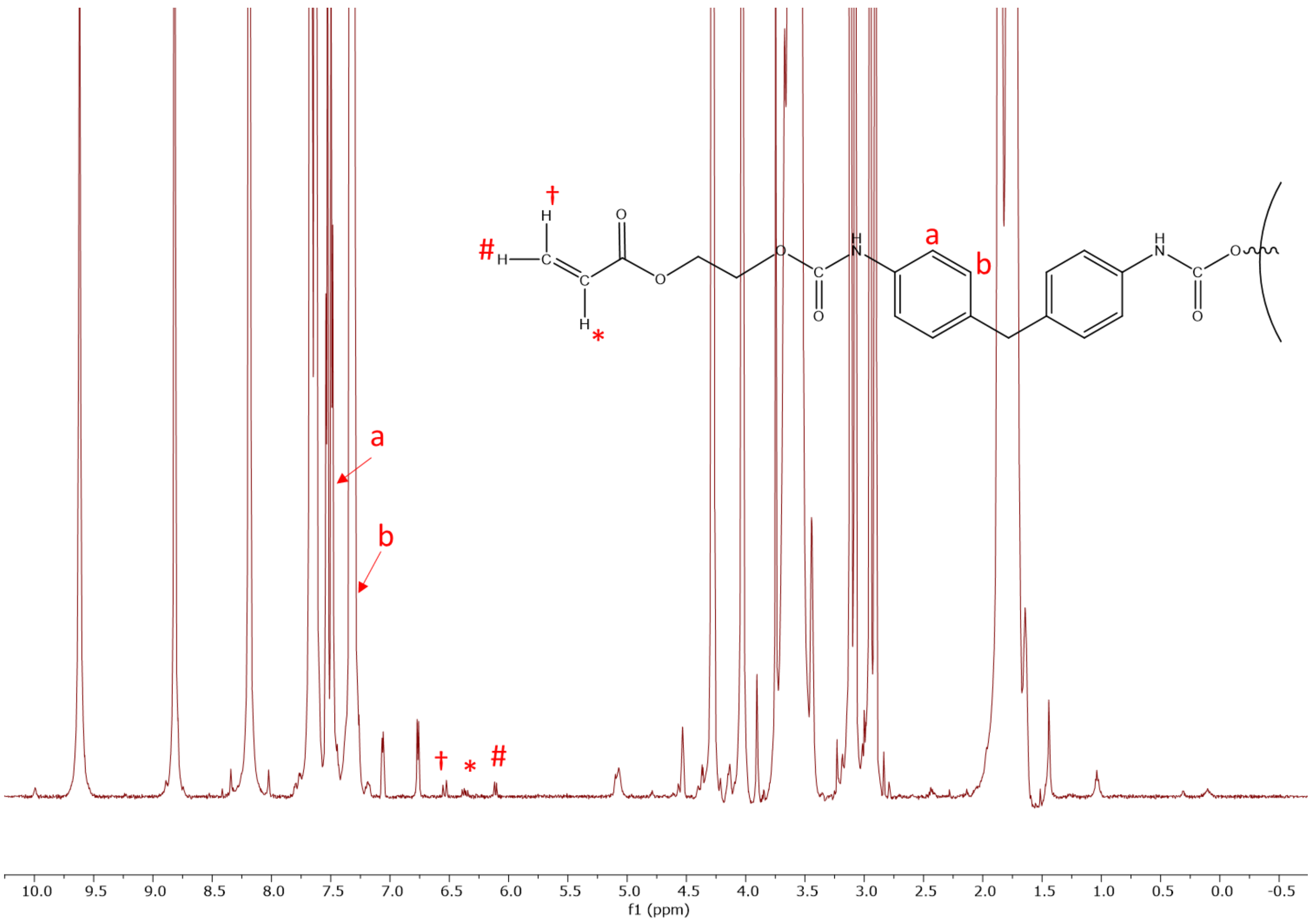

Figure S2. ^{1}H NMR spectra of PUDA-PQ in DMF-d_7.

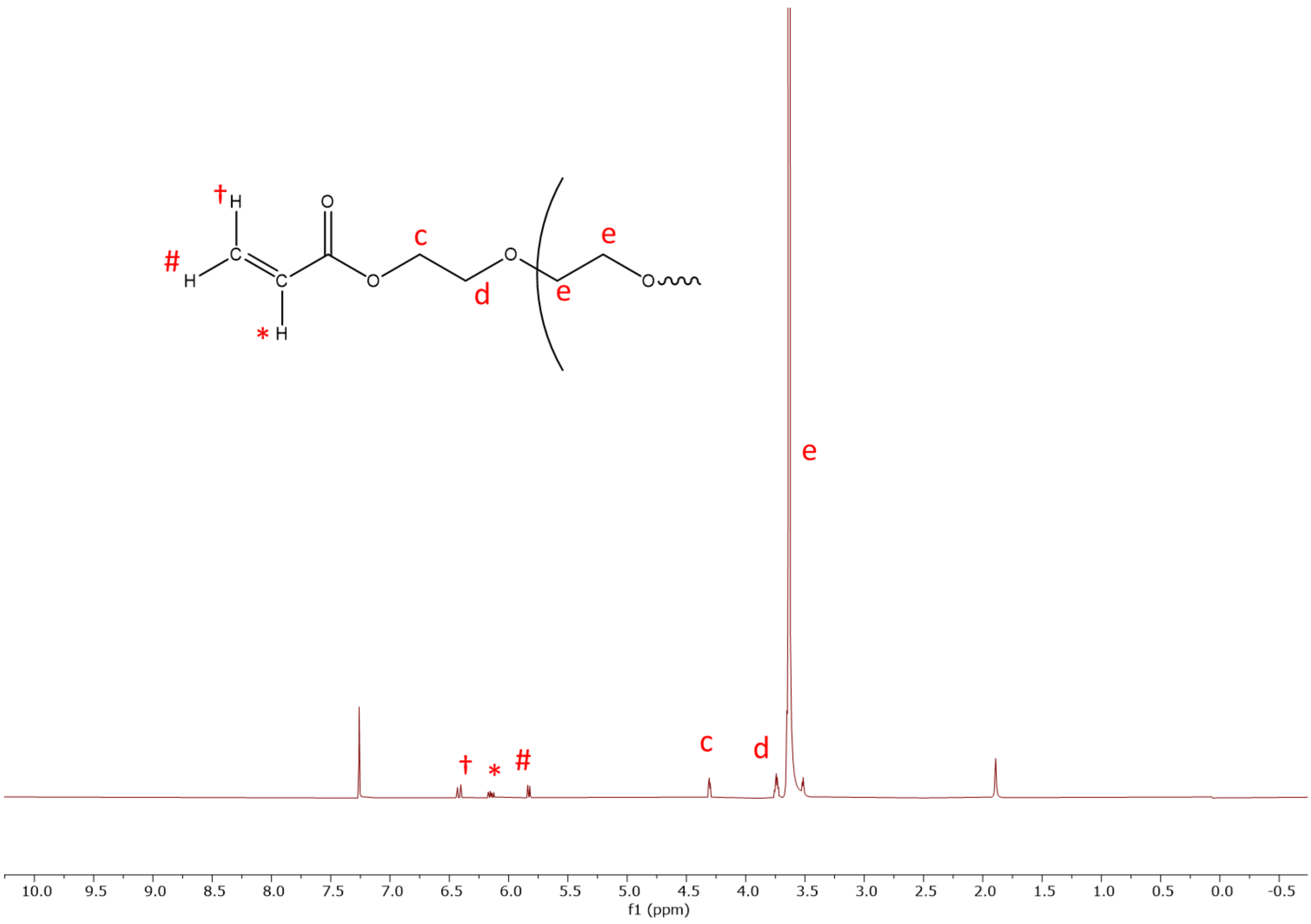

Figure S3. ^{1}H NMR spectra of PEGDA in chloroform-d.

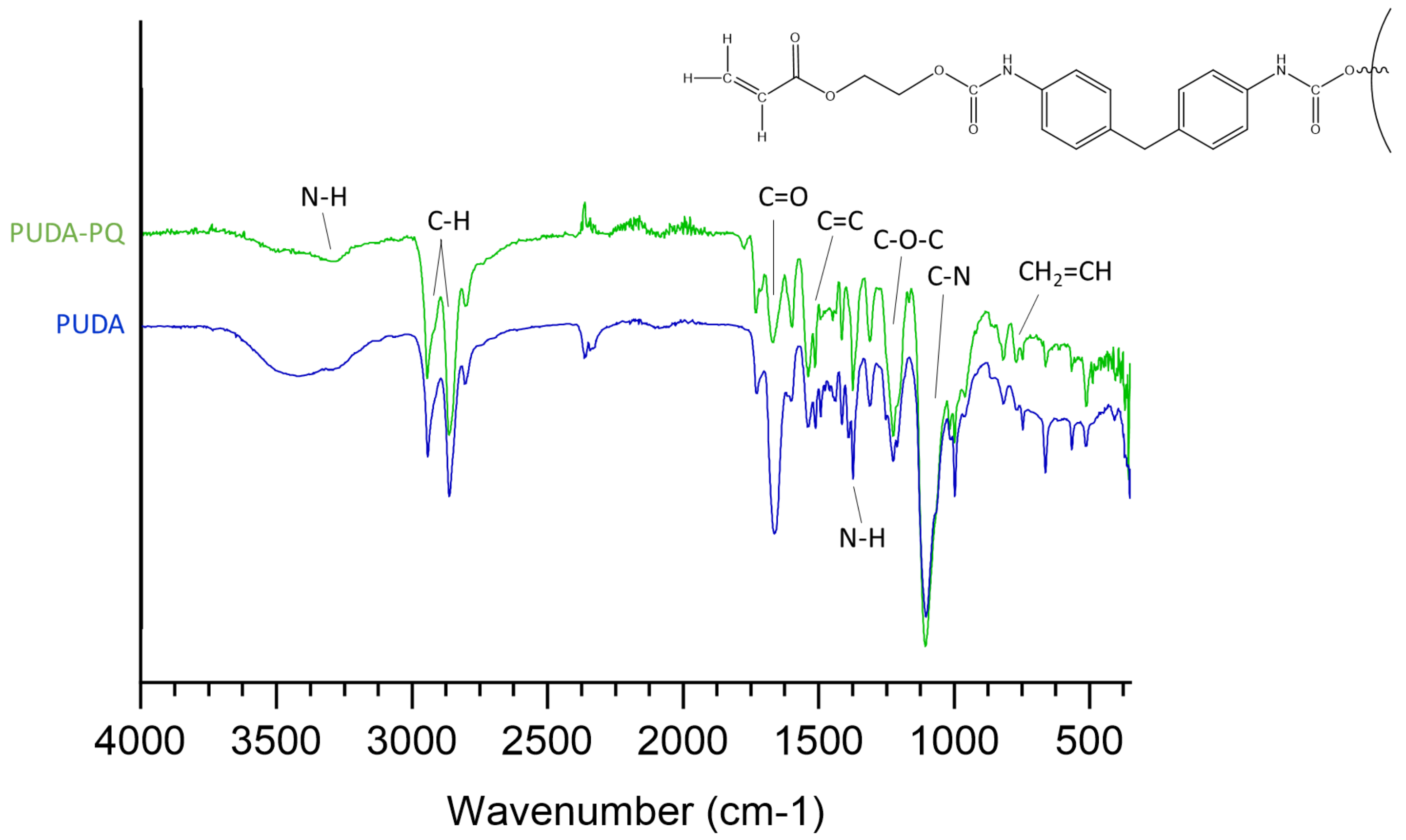

Figure S4. FTIR spectra of PUDA and PUDA-PQ.

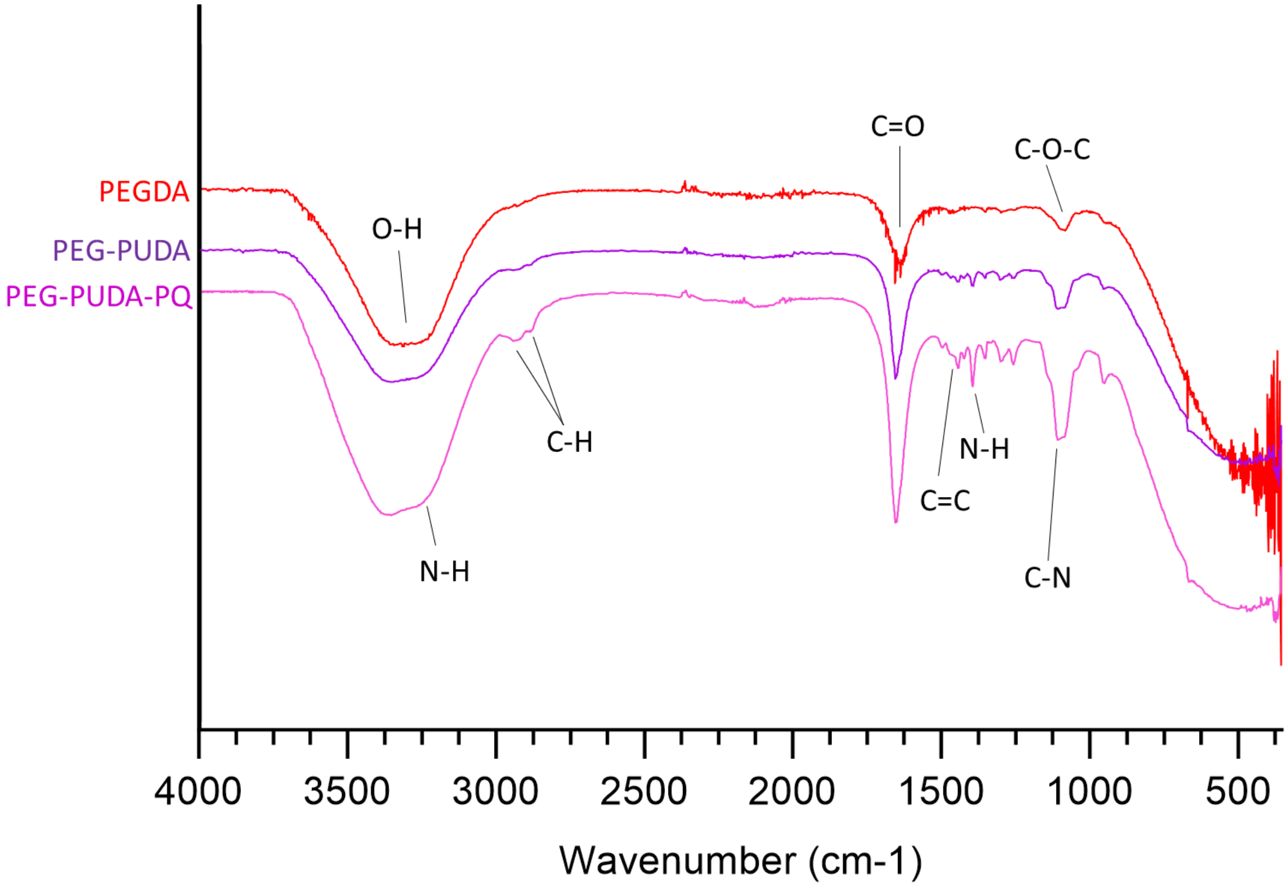

Figure S5. FTIR spectra of PEGDA, PEG-PUDA, and PEG-PUDA-PQ hydrogels.

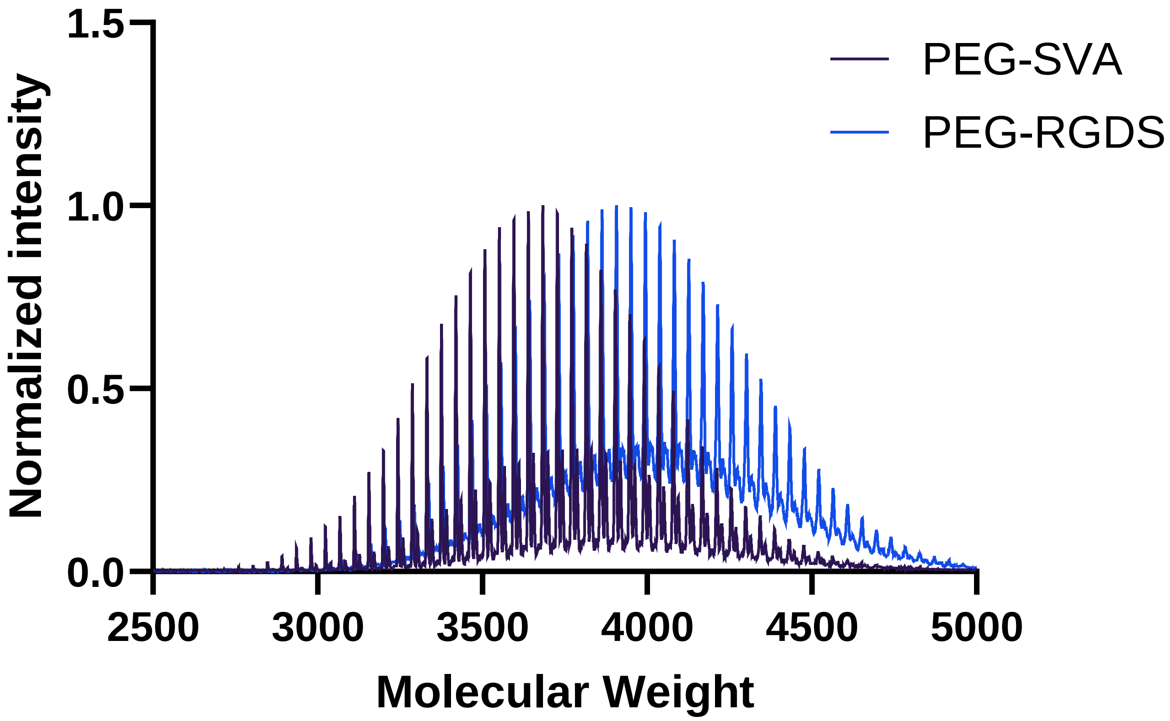

Figure S6. MALDI spectra of acrylate-PEG-SVA and acrylate-PEG-RGDS. Successful conjugation of the RGDS peptide to acrylate-PEG-SVA results in a shift in molecular weight.

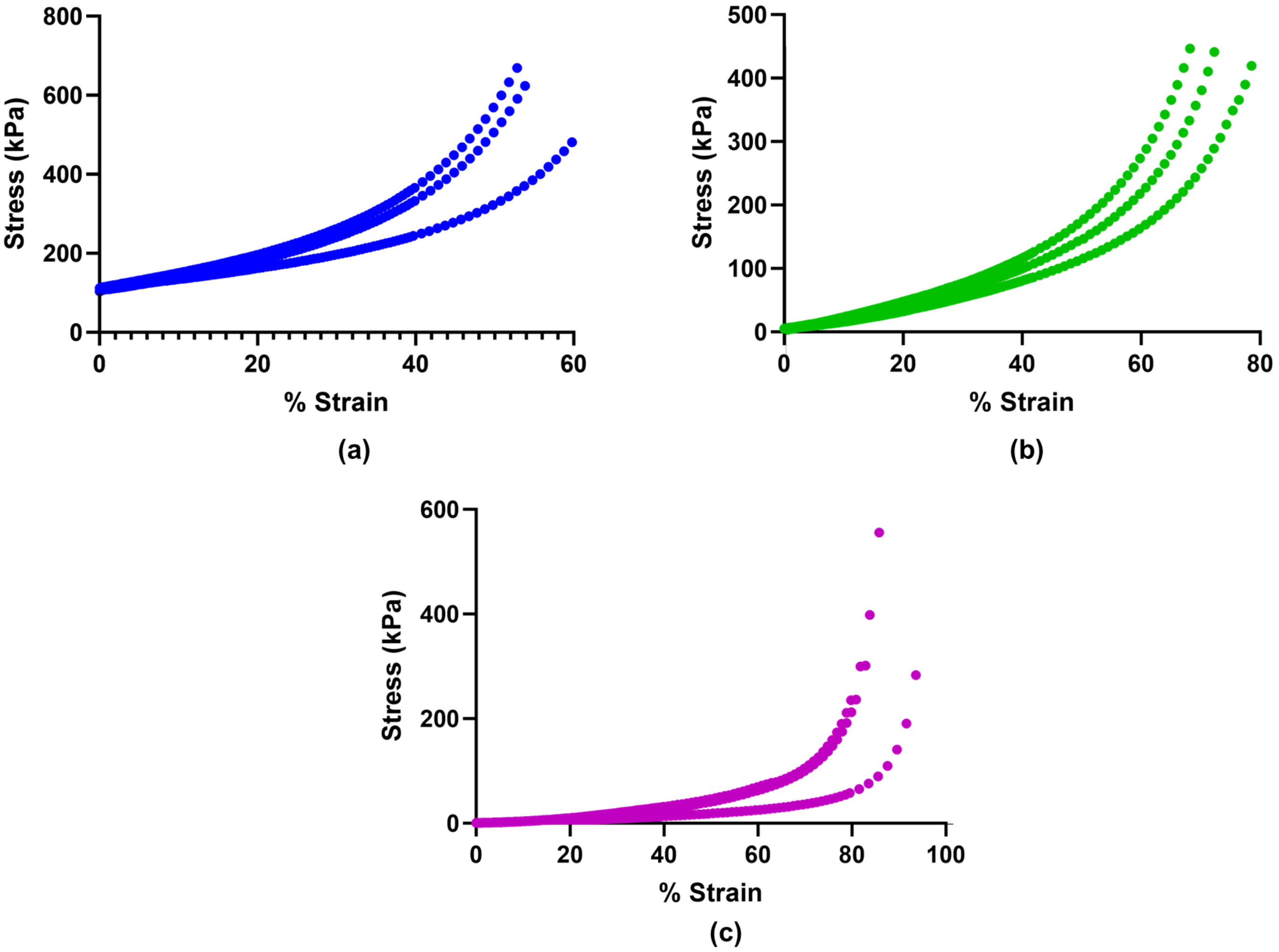

Figure S7. Stress-strain curves for a) PEGDA hydrogels, b) PEG-PUDA hydrogels, and c) PEG-PUDA-PQ hydrogels. n = 3 samples per condition. The compressive modulus was calculated as the average slope of the linear region following the toe region for each data set.

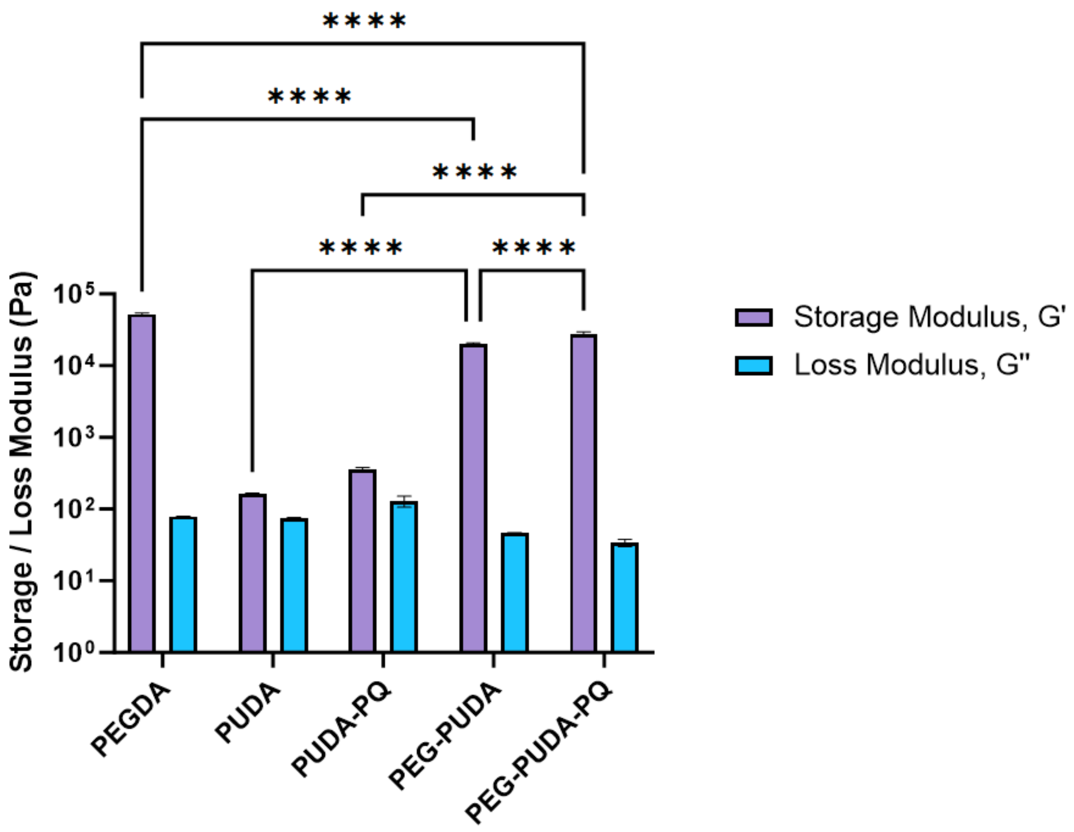

Figure S8. Storage and loss modulus for 10% w/v PEGDA, acrylate polyurethane films (10% w/v), and PEGDA-polyurethane hydrogels (containing 10% w/v PEGDA and 10% w/v PU). Error bars indicate standard deviation of the mean of 3 UV-cured samples per condition (n = 3); ****$p < 0.0001$.

Table S1. Rheology data from hydrogels cured during oscillatory shear time sweeps (1 Hz, 1% strain) with a 2 min UV exposure (365 nm, 10 mW/cm^2). Data represent the mean value of 3 hydrogel samples (n=3).

Properties	PEGDA	PEG-PUDA	PEG-PUDA-PQ
Storage Modulus (Pa)	52175.67±2167.82	20052.33±724.58	27690±2120.42
Loss Modulus (Pa)	79.14±1.01	46.38±0.61	33.90±3.99
Complex Viscosity (mPa·s)	$8.30\times10^6 \pm 0.35\times10^6$	$3.17\times10^6 \pm 0.11\times10^6$	$4.41\times10^6 \pm 0.34\times10^6$
tan δ	$2.34\times10^{-3} \pm 0.69\times10^{-3}$	$1.22\times10^{-3} \pm 0.41\times10^{-3}$	$2.24\times10^{-3} \pm 0.35\times10^{-3}$

Table S2. Swelling ratios and percent mass loss of hydrogels and thin PU films. Data represent the mean value of 4 samples (n=4).

Sample	Swelling Ratio	% Mass Loss
PUDA	0.213±0.109	0.69±1.75
PUDA-PQ	0.213±0.060	-0.11±2.82
PEGDA	1.903±0.142	1.17±2.06
PEG-PUDA	1.39±0.070	-1.74±2.82
PEG-PUDA-PQ	1.617±0.053	0.04±2.57
